# Duplicated Anterior Choroidal Arteries: Literature Review and Clinical Implications

**DOI:** 10.7759/cureus.16291

**Published:** 2021-07-10

**Authors:** Michael Artov, Joe Iwanaga, Melanie L Korndorffer, Aaron S Dumont, R. Shane Tubbs

**Affiliations:** 1 Department of Neurosurgery, Tulane University School of Medicine, New Orleans, USA; 2 Department of Structural and Cellular Biology, Tulane University School of Medicine, New Orleans, USA

**Keywords:** blood supply, brain, intracranial, vasculature, variations

## Abstract

The anterior choroidal artery supplies important cerebral structures. One important variation of this vessel is duplication. However, little is reported on this anatomical variant and moreover, the prevalence of such a finding varies widely. Therefore, here, we review the literature regarding duplicated anterior choroidal arteries. Clinicians reviewing imaging of the brain, interventionalists, or neurosurgerons should be knowledgeable of variations of the anterior choroidal artery, including its duplication. A better understanding of this anatomy and embryology can improve diagnoses and patient outcomes following interventional or open neurosurgical techniques.

## Introduction and background

**Figure 1:**
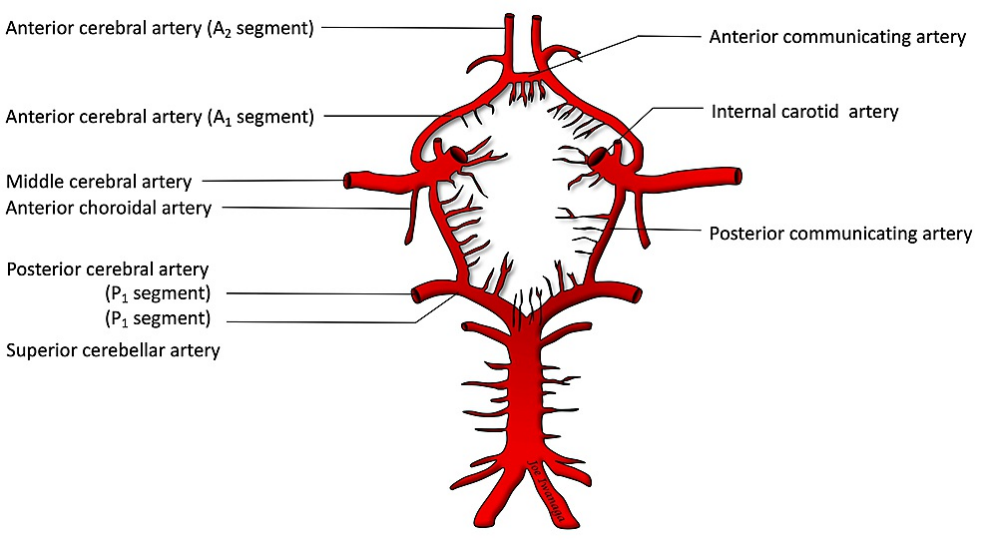
Circle of Willis drawing

The human brain receives a complex vascular supply. The primary contributors to the anterior circulation of the brain are the internal carotid arteries (ICA) and their corresponding branches [1,2]. Among the branches are the posterior communicating artery (PCom), anterior cerebral artery (ACA), middle cerebral artery (MCA), and the anterior choroidal artery (AChA) (Figure 1).

The AChAs serve several vital structures in the brain responsible for vision and motor function. They branch from the ICA and commonly originate distal to the PCom. The AChAs pose concerns for neurosurgeons because of their tendency to develop aneurysms and potential to exhibit complex anatomical variations, including course, size, segments, branching patterns and brain regions supplied by these vessels [3-7]. The aim of this paper is to review surgical anatomy, embryology, anatomical variations, pathology of the AChA and its duplication.

## Review

Surgical Anatomy 

The perforating branches of the AChAs can be classified by their surgical areas of interest or in terms of their primary segments, the cisternal (extraventricular segment) and the plexal (intraventricular segment) [1-9]. Generally, the cisternal segment begins at the origin of the AChAs (the ICA), courses along the optic tract, travels through the temporal lobe, and terminates medially to the choroid fissure. Once the AChAs enter the choroid fissure, they terminate as the plexal segments and supply the choroid plexus on the temporal horn. In their perfusion, the AChAs are typically restricted to: (1) a temporal choroid branch to the choroid glomus, (2) deep perforators to the optic tract, posterior limb of the internal capsule and deep white matter, and variable portions of the adjacent medial globus pallidus and lateral thalamus (3) anterior hippocampal, parahippocampal, and uncal cortices [9]. The size and structures supplied by these vessels can be variable as observed in Hussein et al. and increases the importance of these arteries during surgery [4]. In fact, Malis et al. [10], Wilkins et al. [11], and Yaşargil et al. [12,13] make note that the AChA and perforating branches may be found in the surgical area of sphenoid ridge tumors, pituitary adenomas, sellar and parasellar tumors, and anterior circulating and basilar tip aneurysms [5]. Due to their role in supplying vital structures in the brain, the integrity of the AChAs and their branches is important.

Embryology

Observed variations in the AChAs may be due to their extensive vascular demand throughout early stages of brain development. The AChAs are early-developed arteries and supply large portions of the brain during week five of embryonic development, also known as the choroidal stage of development [1,7,9]. Around this stage, the AChAs branch from the anterior division of the early ICA, make their way behind the neck of the growing cerebral hemisphere, and supply large portions of the developing diencephalon and telencephalon [3,9]. It is also stressed by Raybaud [9] that the AChAs feed the inferior segment of the meninx primitiva, the early precursor to the choroid plexus. The choroid plexus plays a vital role in supporting early brain tissue as the ventricles begin to develop in the following weeks. This well vascularized region of the developing brain is highly metabolically active and places a heavy load on its arterial feeders: the ACA, the posterior choroidal artery posteriorly (PChA), and the AChAs inferiorly [9] Conditions surrounding this initial meshwork consolidation and artery formation may explain the variants and morphological abnormalities observed in adult AChAs [1,9] Later in brain development, the AChAs commonly regress in their prominent perfusion (relinquished to the growing posterior cerebral artery) and mostly supply the choroid plexus of the forebrain and the temporal uncus.

Variations

Anatomical studies of the AChA dealing with its course and regions supplied have been studied extensively. However, much anatomical variation has been described, leading authors to make clear distinctions in their definition of the AChA [1-11]. These variants have warranted thorough anatomical review of the ICA and AChA prior to considering neurosurgical intervention and, in some cases, such as those described in Chenin et al. [2], have drawn attention to their potential risk for aneurysms. 

In adults, the fully developed AChAs commonly originate from the ICAs and have multiple perforating branches with important defining characteristics. Although rare, variations in the origin of the AChAs and branching pattern are observed. In respect to their origin, it is common to identify the AChAs by their direction off the ICA and relationship to the PCom [1-11]. In a large intraoperative study performed by Akar et al. [5], 130 patients were operated via a pterional approach to observe AChA variation; the AChAs arose from the inferolateral aspect of the ICA in 95 cases (73%), the posterolateral aspect in 27 cases (20.7%), and from the lateral aspect in 8 cases (6.3%). Following a cadaveric study performed by Uz et al. [7], the authors reported the AChAs arising from the posteroinferior, posterolateral, and anterior aspect of the ICA at a rate of 60%, 27%, and 13% respectively and on average 5.3 mm distal to the PCom and 4mm proximal to the carotid bifurcation. In a separate cadaveric study, Hussein et al. [4] define the AChAs as those arteries arising from the inferolateral wall of the ICA, 3.2 mm distal to the PCom, and 5.2 mm proximal to the carotid bifurcation. It is worth mentioning that Saeki and Rhoton, Uz et al., and Akar et al. reported having all AChAs originate from the ICA [5-7]. This is in stark contrast to the findings reported in the literature reviews performed by Hussein et al. and Saeki and Rhoton where numerous variations in the origin of the AChAs were described [4,7]. The variants reported include origins arising from the bifurcation of the ICA, MCA, and PCom. 

Pathology

The AChAs are involved in many pathologies and diseases including brain infarcts, brain tumors, Moyamoya disease, arteriovenous malformations, and aneurysms [1,8]. Therefore, understanding the clinical features associated with damage to these arteries is essential for establishing a diagnosis and may even suggest primary preventative applications. Pathology of the AChAs produces classic AChA syndrome with clinical features of contralateral hemiplegia, hemisensory loss, and homonymous hemianopia [1,2,5,7,14]. This syndrome is, however, often rare and incomplete. In a prospective study performed by Ois et al. [14] 1350 patients with acute ischemic stroke were evaluated to describe the prevalence, prognosis, and common symptoms associated with AChA infarcts. The study detected 112 patients (8.3%) with AChA infarcts; the most common symptoms associated with the AChA infarcts were contralateral motor weakness and sensory dysfunction; the complete classic syndrome (hemiparesis, hemianesthesia, and hemianopia) was registered in 12 patients (10.7%) [14]. Given the complexity of the clinical features associated with AChA damage, a further look into the microvasculature may be necessary when establishing a diagnosis, especially given the numerous pathologies associated with the AChAs. 

Although AChA aneurysms account for approximately 4% of all intracranial aneurysms, they are the most common lesion involving the AChAs [1,2]. Aneurysms of this artery are generally located near its origin, making this area a point of interest for surgeons [5]. 

Anterior Choroidal Artery Duplication

**Figure 2:**
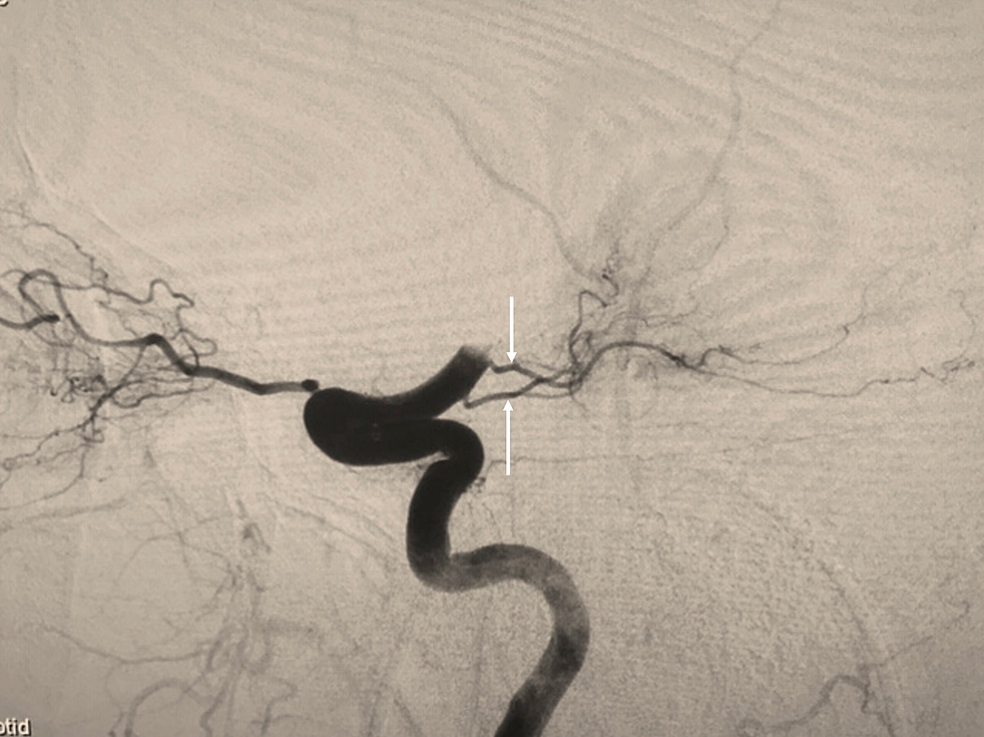
Patient evaluated with angiography to evaluate for acute stroke and noting duplicated anterior choroidal arteries (arrows).

Duplicated AChAs are rare and/or underreported [2, 4-6] (Figure 2).

During our literature review of anatomical variations of the AChA, we collected data on the frequency of duplicated AChA. Chenin et al. described a duplication of the right AChA, with an aneurysm located at the branch’s origin [2]. Double origin anterior choroidal arteries were reported in 4%, 4.5%, and 13% by Saeki and Rhoton, Hussein et al., and Akar et al., respectively [4-6]. Aneurysms among AChA duplications have been reported [2,3]. These findings, along with those described in this case may pose interesting clinical and embryological implications and invites further investigation into the anatomy of the AChA and its variations. Interestingly, Morandi et al. [15] have posited that some of the reported AChA duplications might be uncal branches of the normal AChA. If this is true, the prevalence of true duplications might be much less that currently reported in the literature.

## Conclusions

Clinicians reviewing imaging of the brain, interventionalists, or neurosurgerons should be knowledgeable of variations of the anterior choroidal artery, including its duplication. A better understanding of this anatomy and embryology can improve diagnoses and patient outcomes following interventional or open neurosurgical techniques.
